# Overexpression of Trps1 contributes to tumor angiogenesis and poor prognosis of human osteosarcoma

**DOI:** 10.1186/s13000-015-0401-2

**Published:** 2015-09-17

**Authors:** Zhishuang Li, Ming Jia, Xiaojuan Wu, Jingjing Cui, Aifeng Pan, Li Li

**Affiliations:** Department of Pathology, Shandong University, School of Medicine, 44 Wenhua Xi Road, Jinan, Shandong 250012 People’s Republic of China; Shandong University, School of Medicine, 44 Wenhua Xi Road, Jinan, Shandong Province 250012 People’s Republic of China

## Abstract

**Background:**

Trichorhinophalangeal syndrome 1 (Trps1) gene is a member of GATA transcription factor family and has an important function in tumorigenesis and progression. However, there are rare studies on its roles in carcinogenesis and prognostic significance in human osteosarcoma.

**Methods:**

The expression of Trps1 was detected by immunohistochemistry, and MVD was evaluated to determine the amounts of microvessels by counting CD31-positive endothelial cells.

**Results:**

Of the 74 cases that underwent study, Trps1-positive cases were 24. And it was associated with MVD significantly (*P* = 0.008). The data also exhibited more cases of remote metastasis (*P* = 0.013) and higher Enneking stage (*P* = 0.017) in Trps1-positive group compared to Trps1-negative group. Univariate analysis revealed that distant metastasis, MVD and Trps1 expression were associated with a lower 3-year overall survival rate and disease-free survival rate (*P* = 0.003, and *P* = 0.012 respectively). Furthermore, Trps1 and distant metastasis retained their significant prognostic effects on patients survival rate by multivariate analysis (*P* < 0.05).

**Conclusions:**

Trps1 plays a crucial role in osteosarcoma angiogenesis, metastasis and clinical surgical stage. Trps1 can be a novel promising prognostic marker and therapeutic target, and antiangiogenic therapy which targets Trps1 molecule in patients with osteosarcoma may lead to improved prognosis and longer-term survival.

## Background

Osteosarcoma is the most common primary malignant bone tumors occurring in adolescents and young adults [1–3]. Although various treatments such as wide excision surgery of tumors, radiotherapy, chemotherapy as well as neoadjuvant chemotherapy have made significant improvements in the long-term outcome of these patients [4, 5], it is still not satisfactory. There are still 30-40 % of children dying of osteosarcoma, and 25 ~ 50 % of patients subsequently develop metastatic disease, which remains the major cause of death [6]. Presence of primary metastasis has been proved to be an independent prognostic indicator in osteosarcoma [7].

The process of metastasis consists of a series of complex procedures in which sustained angiogenesis plays a crucial role [8–11]. Tumor angiogenesis depends on the proliferation, migration and attachment of vascular endothelial cells, and is regarded as a hallmark of cancer. It has been clear that there is a significant correlation between angiogenesis and prognosis in many cancers, including gastric, colorectal, breast and prostatic cancers [12–15]. Several studies also have revealed high metastatic potential and recurrence rate of osteosarcoma are associated with high levels of angiogenesis [16, 17]. Anti-angiogenic therapy which targets vascular growth within tumors with the aim of suppressing tumor growth and metastasis is now widely approved to treat different tumors. Anti-angiogenic reagents theoretically have fewer side effects, because neoangiogenesis rarely occurs in healthy adults.

Trps1 is a gene encoding a new member of GATA family of transcriptional regulators and represses transcription of genes containing -GATA- sequences. It consists of nine zinc-finger domains, including a GATA-type zinc finger through which it binds DNA. Either mutation or deletion of this gene causes a disease called Tricho-rhino-phalangeal syndrome (TRPS) clinically which is characterized by craniofacial and skeletal abnormalities. Previous studies have proved that Trps1 is expressed in several human malignant tumors and implied an important function in tumor growth, invasion, and metastasis [18–20]. Furthermore, the abilities of chondrogenesis and apoptosis in ATDC5 cells were enhanced by Trps1 [21]. And Trps1 has been shown to play pivotal roles in the development of bone. Recently, it has been exhibited that elevated Trps1 expression promoted angiogenesis by affecting the expression of vascular endothelial growth factor (VEGF) in breast cancer [22]. Hence, this current study was designed to explore Trps1 expression and evaluate its significance to intratumoural microvessels density in osteosarcoma. Their associations between Trps1 and clinicopathological variables and prognosis were evaluated further.

## Methods

### Patients

A retrospective analysis showed that there were 157 patients who presented with osteosarcoma histopathologically between March 2004 and August 2010 at the Department of orthopedic surgery, Qilu Hospital of Shandong University. Among them, in order to avoid interfering, patients who did not receive neoadjuvant treatment prior to diagnostic biopsy and whose paraffin-embedded tissues are available for immunohistochemical analysis were included. 74 eligible patients who underwent tumor surgical resection and reconstruction or amputation were identified for this study. Clear margins were achieved in all cases. These patients consisted of 51 males and 23 females, and the age range was from 11 to 54 years (median 25 years). Histologically, these osteosarcoma samples included 62 cases of conventional type (42 osteoplastic, 13 chondroblastic and 7 fibroblastic type respectively) and 12 cases of special type (5 parosteal, 1 periosteal, 5 low grade central and 1 telangiectatic type). Tumor staging was evaluated based on the Enneking surgical classification. 11 cases were at stageI, 58 at stage II, 5 at stage III. There were 7 cases of recurrence, and 31 cases of distant metastasis comprising 5 of pre-surgical metastasis and 26 of post-operative metastasis. In detail, the clinicopathological parameters of these 74 cases were listed in Table 1. This study was approved by the Ethics Committee of Qilu Hospital and previous informed consent was obtained from all patients.

**Table 1:** Correlation of clinicopathological variables with Trps1 in human osteosarcoma

Variables	Numbers	Trps1		*P*
		negative	positive	
Sex				0.434
male	51	33	18	
female	23	17	6	
Age				0.628
<20	34	22	12	
≥20	40	28	12	
Size				0.100
<8 cm	41	31	10	
≥8 cm	33	19	14	
Differentiation				0.091
well	11	10	1	
poor	63	40	23	
Position				0.329^a^
femur	37	22	15	
tibia	16	12	4	
others	21	16	5	
Type				0.089^a^
common	62	39	23	
special	12	11	1	
Metastasis				0.013
yes	31	16	15	
no	43	34	9	
Enneking Stage				0.017^b^
I	11	10	1	
II	58	39	19	
III	5	1	4	

^a^Fisher’s exact test ^b^Likelihood ratio

### Immunohistochemical Staining for Trps1 and CD31

Continuous sections in 4 μm thick were prepared from each formalin-fixed, paraffin embedded tissue. Immunohistochemical staining was performed to evaluate the expression of Trps1 and CD31.

All sections on the slides were dewaxed, and rehydrated with xylene and graded alcohol, then dripped 3 % hydrogen peroxide on them to quench endogenous peroxidase. Afterwards, high-temperature antigen retrieval was carried out in citrate buffer (pH 6.0) in a microwave oven to enhance immunoreactivity, followed by 5 % normal horse serum to reduce the non-specific bindings. Primary antibody against Trps1 (sc-26974, Santa Cruz Biotechnology, Inc. USA, 1:200) and CD31 (Zhongshan Biotechnology Company, Beijing, China) were applied to the sections respectively and incubated overnight at 4 °C. Subsequently, slides were incubated with the biotinylated second antibodies and streptavidin–peroxidase conjugate, and antibody-specific binding was visualized with 3, 3-diaminobenzidine solution (DAB). Lastly, slides were counterstained with hematoxylin and mounted. PBS was used as a negative control by replacement of the relevant primary antibody.

### Evaluation of Trps1 expression and MVD

Trps1 showed nuclear staining, and scoring was based on a semi-quantitative scoring system according to both the staining intensity (0, no staining; 1, weak; 2, moderate and 3, intense staining) and percentage of positive tumor cells on a 4-point scale as follows: 0, <5 % positive cells; 1, 5 % ~ 25 % positive cells; 2, 26 % ~ 50 % positive cells; 3, >50 % positive cells. The final staining score was the multiplication of scores of staining intensity and percentage of positive cells. Cut-off levels were further applied as follows: 0 (−); 1–3 (+); 4–6 (++) and 7–9 (+++). So the expression of Trps1 was divided into a non-overexpression group (− or +) and an overexpression group (++ or +++). Tumor microvessels were recorded by counting the CD31-positive stained endothelial cells. MVD was assessed according to a modified version of the International Consensus Report [23]. Briefly, the immunostained sections were initially scanned at a low power (100 × magnification) to identify “hot spots”, which are the areas with the most intense vascularity. Subsequently, counting of the stained microvessels was performed on three consecutive high power (200 × magnification) fields within “hot spot”. Any yellow-brown immunostained endothelial cells or endothelial cell cluster that was clearly separate from adjacent microvessels could be considered as a single countable micovessel [13]. The average of three 200 × field microvessel counts was captured as the value of MVD, and the cut-off value for categorical evaluation of MVD was predefined as the median microvessel count: Tumors with microvessel numbers ≥ median value were defined to be high MVD group, while < median value were defined to be low MVD group. Immunohistochemical analysis and scoring were performed by two investigators (LL and XJW) who were blinded to the diagnosis, clinical course and outcome of patients independently.

### Follow-up

Follow-up period was defined from hospital discharge to the date of patient’s death or the last follow-up, and data were collected either on an outpatient basis or by telephone interview. The living status was confirmed, and the median period of follow-up was 40 months (range, 4–82 months). Overall survival (OS) was calculated from the date of surgery to the date of death. Disease-free survival (DFS) time was defined as the interval between the date of surgery and the date of recurrence. If recurrence was not diagnosed, the survivors were censored on the date of death or the last date of follow-up. Data involving the conventional clinicopathological parameters also were collected for analysis, including gender, age, tumor size, differentiation, position, metastasis and surgical stage.

### Statistical Analysis

Statistical analyses were carried out by using the software package SPSS 19.0 for Windows (SPSS Inc. Chicago, USA). The Chi-square test was used for statistical analysis between Trps1 and categorical data. Survival curves were constructed through the Kaplan-Meier method and were evaluated using the log-rank test. *P* values that less than 0.05 were considered to be statistically significant.

## Results and Discussion

### The expression of Trps1 and the correlation with MVD

**Fig. 1:**
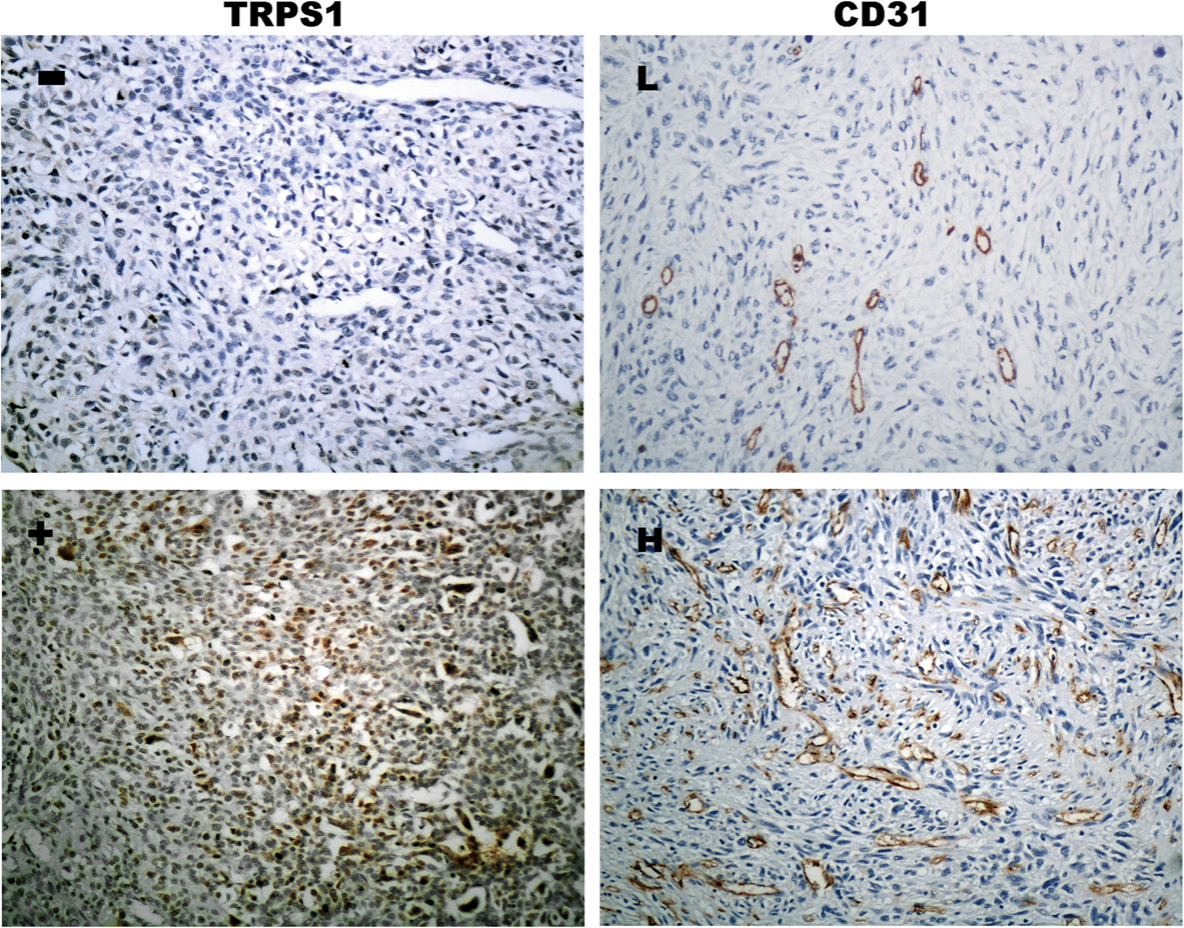
Trichorhinophalangeal syndrome type 1 (Trps1) and CD31 expression in osteosarcoma tissues by Immunohistochemical staining. CD31 indicates intratumoural microvessels. Representative Fields showed the negative outcome of Trps1 and CD31 (upper row, ×200) and the positive outcome (lower row, ×200). Trps1-positive staining was observed in nucleus. The cut-off value of microvessels was 41/×200 field

The expression of Trps1 was detected in 24 of 74 (32.4 %) cases, whereas 50 (67.6 %) cases showed negative staining in tumor samples. Representative immunostaining with different intensities were shown in Fig. 1. In order to evaluate the relationship between Trps1 and angiogenesis, microvessels were stained by CD31. Analysis of CD31 immunostained sections demonstrated that the median MVD was 41 microvessels (range, 14 to 79). High MVD (≥41 microvessels) was observed in 39 out of 74 cases (52.7 %). There were 18 Trps1-positive (75 %) and 21 Trps1-negative (42 %) samples respectively in high MVD group. Therefore, it showed a significant correlation of Trps1 expression with MVD (*P* = 0.008). The Spearman correlation analysis further proved these results (r = 0.355, *P* = 0.002, Table 2).

**Table 2:** The correlation of Trps1 protein with MVD

Trps1	Numbers	MVD		*P* value^a^	Spearman	Value (r)	*P* value^b^
		High	Low		correlation		
Positive	24	18	6	0.008		0.355	0.002
Negative	50	21	29				

^a^Chi-square Test

^b^The Spearman correlation was used to compare the degree of correlation. Positive numbers reflected directed correlation

### Correlation of Trps1 expression with clinicopathological variables in human osteosarcoma

We analyzed the effect of Trps1 expression on different clinicopathological variables. As shown in Table 1, these data exhibited the expression of Trps1 was not associated with gender, age, tumor size, position, type and differentiation (*P* > 0.05), but related with metastasis (*P* = 0.013) and Enneking Stage (*P* = 0.017) significantly. Patients with Trps1-positive expression had a higher tendency of both distant metastasis and a further clinical stage.

### The correlation of Trps1 expression with the prognosis in patients with osteosarcoma

**Fig. 2:**
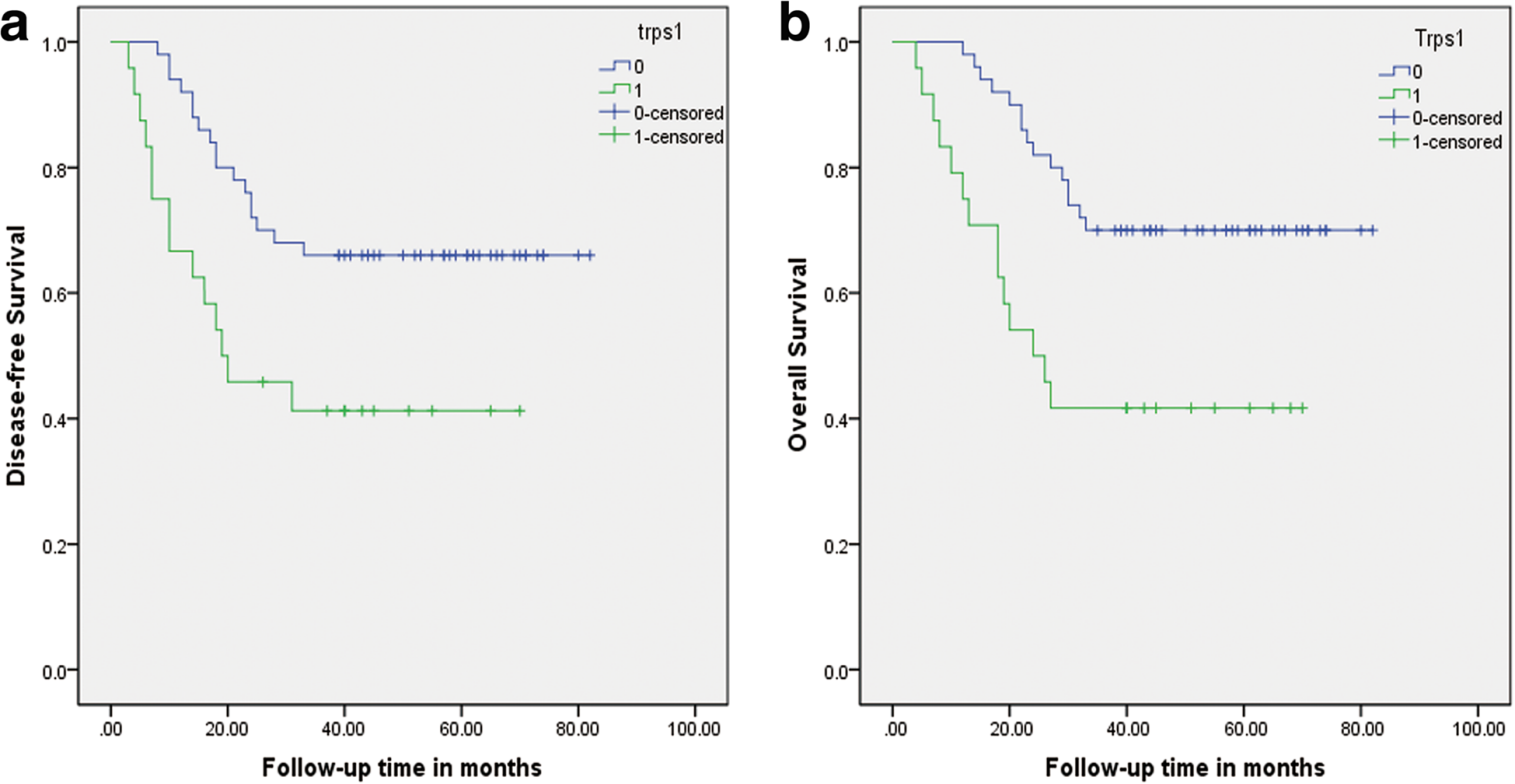
Different Kaplan-Meier curves of overall and disease-free survival curves stratified in terms of the differential expression of Trps1. Figure 2a showed that there was a significant difference in 3-year DFS rate between Trps1-positive and Trps1-negative (41.7 % vs 66 %, *P* = 0.012). Figure 2b showed a worse 3-year OS rate in Trps1-positive group (41.7 % vs 70.0 %, *P* = 0.003)

To provide a powerful explanation of the prognostic role of Trps1, we assessed the effects of Trps1 expression on the 3-year OS and DFS rates by Kaplan-Meier survival analysis. In all 74 patients, tumor recurrence developed in 31/74 (41.9 %), including distant metastasis in 24 cases, local recurrence in 5 cases and both in 2 cases. Separately, tumor recurrence occurred in 14 out of 24 (58.3 %) cases with Trps1-positive while 17 out of 50 (34.0 %) cases with Trps1-negative. 49 patients died at the end of follow-up period. Figure 2 showed the cumulative OS and DFS curves of all 74 patients which were stratified by Trps1 expression levels. 29 patients (39.2 %) died within 3 years after operations, in which 14 cases (58.3 %) showed Trps1-positive. Kaplan-Meier survival curve exhibited a worse 3-year DFS rate in patients with Trps1-positive expression (*P* = 0.012, Fig. 2b). There was a significant difference in 3-year OS rate between two groups (*P* = 0.003, Fig. 2a).

### Univariate and Multivariate Survival Analysis

Univariate and multivariate analysis were performed to investigate whether Trsp1 could be an independent factor to predict prognosis among sorts of clinicopathological parameters (Table 3). Univariate analysis revealed that distant metastasis, MVD and Trps1 expression were associated with a poorer 3-year overall and disease-free survival rate (*P* < 0.05) but were not associated with gender, age, tumor position, size, histological type and differentiation (*P* > 0.05). Further, with multivariate analysis, Trps1 and distant metastasis retained their significant prognostic effects on survival rate of patients (*P* < 0.05), however MVD didn’t (*P* > 0.05),which indicated the possibility of cross-talk between Trps1 expression and MVD. The influence of MVD on prognosis in Univariate analysis could be caused by Trps1 partly. These data demonstrated that Trps1 affected tumor angiogenesis and could predict survivals for patients with osteosarcoma as an independent prognostic factor.

**Table 3:** Univariate and multivariate analysis of different prognostic parameters

	Univariate analysis		Multivariate analysis			
	P^1^	P^2^	95.0 % CI	P^1^	95.0 % CI	P^2^
gender	0.136	0.570	0.289 ~ 2.623	0.806	0.220 ~ 1.994	0.463
age	0.054	0.097	0.665 ~ 6.486	0.209	0.837 ~ 8.657	0.097
position	0.238	0.054	0.391 ~ 4.007	0.704	0.579 ~ 7.060	0.503
size	0.389	0.602	0.289 ~ 1.744	0.455	0.314 ~ 1.964	0.605
type	0.091	0.068	0.343 ~ 5.942	0.974	0.514 ~ 10.785	0.964
differentiation	0.106	0.094	0.398 ~ 8.899	0.425	0.386 ~ 4.977	0.617
metastasis	0.001	0.001	0.003 ~ 0.082	0.001	0.002 ~ 0.042	0.001
MVD	0.027	0.028	0.821 ~ 5.597	0.119	0.859 ~ 6.252	0.097
Trps1	0.003	0.012	0.115 ~ 0.791	0.015	0.170 ~ 1.108	0.041

*P* ^*1*^Overall survival rate, *P* ^*2*^Disease-free survival rate, *CI* Confidence interval

Though known as the gene involved in TRPS, Trps1 has begun to attract wide attention on its roles in carcinogenesis. Trps1 was first discovered as one of the differentially expressed genes between androgen-dependent and androgen-independent prostate cancer cell lines. Then Trps1 has been implicated in several human cancers, including breast cancer, leukemia, endometrial cancer and colon cancer. However, the roles of Trps1 in tumorigenesis of osteosarcoma are still largely unclear up to now. We investigated the expression of Trps1 in primary human osteosarcoma samples, and observed nuclear stainings of Trps1 in 24 out of 74 cases with heterogeneous express pattern ranging from low to highly intense staining. The expression of Trps1 was associated with distant metastasis and Enneking stage, but not associated with patient age, tumor size, position, histological type, differentiation. It indicated that Trps1 may be involved in tumor genesis and progression of osteosarcoma, although there are contradictory results about the role of Trps1 in predicting tumor metastasis. It was reported that Trps1 counteracted metastasis in tumors [24], while other evidence indicated the high-level expression of Trps1 was significantly associated with higher pathological stage and positive lymph node metastasis [25].

Many studies have directed at determining the role of angiogenesis, one hallmark of tumorigenesis, as well as characterizing the role of several factors in the regulation of microvessels growth [26]. It has been proved that the reason was the imbalance between pro-angiogenic factors and anti-angiogenic factors. As a result of the induction of “angiogenic switch” during tumor development, there is a threshold change between stimulatory and inhibitory influences, in the favor of angiogenesis [27]. Osteosarcoma is one of the most hyper-vascular tumors characterized by active tumor angiogenesis. Tumor angiogenesis plays a pivotal role in osteosarcoma development and progression. Our current study is the first clinical report to assess Trps1 expression in surgically resected osteosarcoma, and also the first study to investigate the role of Trps1 in relation to angiogenesis and prognosis of osteosarcoma. We detected MVD in order to discover whether Trps1-positive expression affects tumor angiogenesis in osteosarcoma. Our results showed that tumors with an over-expression of Trps1 tended to have a higher MVD, which suggests Trps1 may promote progression of aggressive phenotypes by inducting tumor angiogenesis in the development of osteosarcoma. However, tumor angiogenesis involves in multiple factors and steps, so far the specific molecular mechanism of Trps1 promoting angiogenesis in osteosarcoma is still unclear. Therefore, in light of the findings of this current study, further research with large sample size and osteosarcoma cell lines are needed to validate the precise function of Trps1 in the future.

We further analyzed the correlation of Trps1 expression with the post-operation 3-year survival rate. Kaplan-Meier analysis showed after operation Trps1-positive group had a worse both 3-year OS and DFS when compared with Trps1-negative group, suggesting the potential function of Trps1 as a promising prognostic marker for osteosarcoma. In addition, both univariate and multivariate analysis showed Trps1-positive expression retained its prognostic value as an independent prognostic factor for unfavorable survival. Interestingly, the prognosis significance in MVD group by univariate analysis statistically disappeared in multivariate analysis, it told us that tumor angiogenesis is probably not the only pathway to affect prognosis for Trps1. Whereas, Trps1 showed a favorable clinical prognosis in breast cancer through regulating apoptosis and MET [28, 29]. It was not difficult to find that the majority of data were from endocrine-related cancers. More studies from various tumors are needed to elucidate the exact roles of Trps1 in carcinogenesis and progression. On the other hand, Trps1 might exert its two distinct functions at different times during tumor progression by reducing metastasis but at later stages, acting to promote proliferation and thereby worsening prognosis, as demonstrated by Wu et al. Hereby we have the point to add that Trps1 might have distinct functions in individual tumors and depend on the context.

## Conclusions

In summary, based on the above results, we conclude that Trps1 is associated with tumorigenesis, metastasis, surgical stage and tumor angiogenesis in osteosarcoma positively, as well as prognosis and survival. These findings provided important and new information on the metastasis of osteosarcoma. The study indicated that Trps1 may have a clinical potential not only as a promising prognostic marker, but also as a novel therapeutic target in anti-angiogenesis for osteosarcoma. However, validated research with larger sample capability and osteosarcoma cell lines need to be done before Trps1 can be used for clinical decision-making.

## Abbreviations

Trps1: Trichorhinophalangeal syndrome type 1
MVD: Microvessels Density
TRPS: Tricho-rhino-phalangeal syndrome
VEGF: Vascular endothelial growth factor
DAB: Diaminobenzidine solution
OS: Overall survival
DFS: Disease-free survival
